# Metabolomics Profiling and *AKR* Characterization During Paurometabolous Development of *Corythucha ciliata* (Hemiptera: Tingidae)

**DOI:** 10.1093/jisesa/iez117

**Published:** 2019-12-09

**Authors:** Du Li, Youssef Dewer, Cheng Qu, Fengqi Li, Chen Luo

**Affiliations:** 1 Beijing Key Laboratory of Environment Friendly Management on Fruit Diseases and Pests in North China, Institute of Plant and Environment Protection, Beijing Academy of Agriculture and Forestry Sciences, Beijing, China; 2 Hubei Key Laboratory of Insect Resource Application and Sustainable Pest Control, College of Plant Science and Technology, Huazhong Agricultural University, Wuhan, China; 3 Bioassay Research Department, Central Agricultural Pesticide Laboratory, Sabahia Plant Protection Research Station, Agricultural Research Center, Alexandria, Egypt

**Keywords:** *Corythucha ciliata*, metabolomics, aldo-keto reductase, paurometabolous development

## Abstract

The sycamore lace bug, *Corythucha ciliata* (Say) is an invasive pest infesting trees of the genus *Platanus*. Both adults and nymphs damage the foliage of sycamore trees. Nymphs cannot survive in low temperatures; however, the sycamore lace bug overwinters as adults. In this study, we analyzed the metabolite profiles of this pest to determine significantly regulated metabolites during paurometabolous development from nymphs to adults. The identification of metabolites is essential to convert analytical data into meaningful biological knowledge. A total of 62 metabolites were identified using GC-MS. Among them, 29 different metabolites showed differences in content among nymphs, adult females (AF), and adult males (AM). Five of the 29 metabolites, including caffeic acid, D-glucose, D-mannose, glycerol and aminooxyacetic acid, were significantly increased and nine of them were significantly decreased during the developmental stages from nymph to adult. In addition, we identified three novel aldo-keto reductase (AKR) genes that may play a significant role in the control of glycerol biosynthesis. Moreover, the characteristics and expression levels of these genes were analyzed. This study will provide us with the necessary information to improve our understanding of the changes in metabolites in *C. ciliata* during paurometabolous development.

The sycamore lace bug, *Corythucha ciliata* (Say), is a serious pest of sycamores trees (*Platanus* spp.) that originated from the eastern and central regions of North America. It spread to Italy in 1960 (Pellizzari and Monta 1997) and then became widely distributed throughout Europe (Mazzon and Girolami 2000). *Corythucha ciliata* was first recorded in China in 2002 (Li et al. 2007). Furthermore, it has been observed to cause damage to *P. acerifolia* trees in many Chinese cities (Ju and Li 2010). Sycamore lace bug is a paurometabolous insect. Both adults and nymphs feed on the foliage of sycamore trees and cause chlorotic stippling of leaves. More serious infestations can cause early defoliation of trees and lead to tree death (Halbert and Meeker 2001). A recent study observed that lace bug infestations may have serious consequences for human health, including nuisance biting and cutaneous and systemic reactions. (Izri et al. 2015).

*Corythucha ciliata* developmental stages overwinter as an adult under the loose bark of the host tree and can survive extremely low temperatures as low as −30°C (Malumphy et al. 2007, Xia et al. 2007). However, *C*. *ciliata* cannot complete a generation at 16°C, and the minimum survival temperature of nymphs is 10.95°C (Ju et al. 2011). Thus, temperature tolerances of adults and nymphs differ greatly. As nymphs differentiate into adults, unknown are the underlying metabolic processes coordinated with changes in tissues and organs that confer the greater cold tolerance in adults. Fortunately, new insights can be provided by measuring the metabolite changes in biological fluids or tissues to investigate the response of an organism’s metabolism to many stimuli or stresses (Nicholson and Lindon 2008). Therefore, we conducted a metabolomics study to identify *C*. *ciliata* metabolites during paurometabolism.

Aldo-keto reductases (AKRs) represent a superfamily of NADPH-dependent reductases with diverse functions in the reduction of aldehydes and ketones, which is present in nearly all living organisms (Penning 2015). AKRs are mainly monomeric soluble proteins of molecular weights between 30 and 40 kDa and contain a conserved catalytic tetrad of Tyr, Lys, His, and Asp (Davidson et al. 1978, Bennett et al. 1997). Many studies on AKRs have mainly been conducted on mammals, but there is still little known about AKRs in insects (Yamamoto and Wilson 2013, Guo et al. 2014). AKRs participate in a variety of metabolic pathways including the metabolism of glycerol (Jeffery and Jörnvall 1983), which is important for insects to survive in extremely low temperature (Clark and Worland 2008).

In order to study the developmental metabolites of *C*. *ciliata*, we first identified the key compounds of the insect which regulates development and metamorphosis, and then potential genes associated with one of the key differential compounds were identified. Lastly, the sequence characteristics of these genes were analyzed.

## Materials and Methods

### Sampling Collection and Metabolite Analysis

Insect samples were gathered from the leaves of *P. acerifolia* trees planted in the Plant Protection Institute, Chinese Academy of Agricultural Science in Beijing, China, during September 2015. Differentiation between adult females (AF) and adult males (AM) was based on abdominal morphology (Ju et al. 2009). The insects were reared on *P. acerifolia* leaves in a greenhouse under the following conditions: temperature 25 ± 2°C, humidity 50–70%, and a 16:8 (L:D) h photoperiod. Nymphs of 3rd and 4th instars were selected as the targets. Each sample consisted of 300 individuals and was stored at −80°C until further use. There were six biological replicates for each sample. Extractions of samples were performed using glass homogenizers in 750 µl of cold methanol/chloroform (2:1). Then, we added 500 µl ice-cold water to each sample before centrifuging at 2,200 g for 15 min. The resulting supernatant was then transferred to a 1.5 ml tube and dried. Next, using a double derivatization method (Li et al. 2017), we suspended each sample in 40 µl methoxyamin hydrochloride (Sigma–Aldrich, St. Louis, MO) and then shook it for 2 h at 37°C. Then 70 µl N-methyl-N-(trimethylsilyl) trifluoroacetamide (Sigma–Aldrich) was added before 30 min of shaking at 37°C. Finally, each sample was transferred to a GC/MS glass vial for analysis by GC-MS (4D GC×GC-TOF-MS) (LECO, San Jose, CA).

The GC-MS platform was equipped with a DB-5MS capillary column (30 m length × 250 μm inner diameter and 0.25 μm film thickness; J&W Scientific, Folsom, CA) and coated with 5% diphenyl cross-linked with 95% dimethylpolysiloxane. Then, inject of 1 ml of each sample occurred in splitless mode at 230°C and 2 ml min^-1^ helium carrier gas flow rate. The temperature was programmed to begin at 80°C for 2 min, then raised 15°C/min to 330°C, and held at the high temperature for 6 min. The temperature system of the transfer line was 250°C and ion source was 250°C. The system operated in an electron impact mode set at −70 eV. We acquired the mass spectrometry data using the full-scan mode with an m/z range of 70 to 600 at a rate of 20 spectra per second. Data processing was performed using Chroma TOF 4.3X software (LECO Corporation). Metabolites were identified using the LECO/Fiehn Metabolomics Library and an in-house reference compound library. We corrected the concentrations relative to arabinose concentrations, an internal standard, adjusted for sample loss during extraction or injection. Metabolite contents were log2 transformed. The enrichment of metabolites were analyzed on Metabo Analyst 3.0 (available online: http://www.metaboanalyst.ca). Metabolite quantities were compared using ANOVA and Student’s *t*-test in IBM SPSS Statistics software 20. The metabolic and transcriptome data were mapped based on the biochemical pathway integration of the Kyoto Encyclopedia of Genes and Genomes’ (KEGG) *Drosophila* and pea aphids.

### Gene Cloning, Sequence Analysis, and Quantitative Real-Time PCR

The insects (nymphs, AF, and AM) were collected as previously described in section (2.1.). Total RNA was isolated with the RNAqueous-Micro kit (Life Technologies, Darmstadt, Germany) and then used to synthesis cDNA with PrimeScript RT Master Mix (Perfect Real Time) (TaKaRa). AKRs of *C. ciliata* were identified by tBLAST of the *C. ciliata* transcriptome database of the National Center for Biotechnology Information (NCBI) website (https://www.ncbi.nlm.nih.gov/) using known AKRs. Then, the putative AKRs with *E*-values < 1.0E^-5^ were confirmed by BLASTx against the nr database in NCBI. The complete open reading frames (ORF) of AKR1, AKR2, and AKR3 were cloned by PCR according to the sequences in the transcriptome data. Similar amino acid sequences of *C. ciliata* AKR1, AKR2, and AKR3 were detected using NCBI BLAST, and the 28 OBP from 22 different insects were used for phylogenetic analysis, the protein name and NCBI accession numbers were listed in Supp Table S1 [online only]. Phylogenetic analysis was performed by the Molecular Evolutionary Genetics Analysis (MEGA5) software (Tamura et al. 2011), using the neighbor-joining method with bootstrap values based on 1,000 replicates. The annotation of the AKRs was performed using KOBAS 3.0 (http://kobas.cbi.pku.edu.cn/index.php). The qRT-PCR primers were designed using IDT Primer Quest (http://www.idtdna.com/primerquest/Home/Index) and are listed in Supp Table S2 [online only]. Each qRT-PCR contained 10 μl 2× GoTaq1 qPCR Master Mix (Promega, Madison, WI), 10 μM of each primer pair, 100 ng cDNA and nuclease-free water up to a total volume of 20 μl. The qRT-PCR was performed on the ABIPrism7500 (Applied Biosystems, Carlsbad, CA) and the cycling conditions were as follows: 95°C for 2 min, 40 cycles of 30 s at 95°C and 1 min at 60°C. The relative RNA variations were normalized and corrected to the level of the *actin* gene (NCBI accession number: KX108734). Each experiment contained three biological replicates with three technical replicates. The relative expression level of each sample was calculated by the comparative 2^-ΔΔCt^ method (Pfaffl 2001).

## Results

### Metabolomics of *C. ciliata* in Development

We used GC-MS to analyze the metabolites of nymphs, AM and AF of *C. ciliata*. A total of 62 metabolites were identified (Supp Table S3 [online only]), consisting of 23 amino acids, 13 organic acids, 2 free fatty acids, 1 fatty acid methyl ester, 1 intermediate acidic metabolite, 5 sugars, 2 polyols, 3 phosphates, 2 ureides, and 10 other metabolites. The 62 metabolites were primarily enriched in ten pathways (Table 1). The most abundant in numbers of different metabolites was protein biosynthesis, followed by urea cycle and ammonia recycling.

**Table 1.** Metabolite pathway enrichment analysis

	Total	Expected	Hits	Raw p	Holm p	FDR
Protein biosynthesis	19	1.38	18	4.21E-21	3.36E-19	3.36E-19
Urea cycle	20	1.46	10	2.08E-07	1.64E-05	8.32E-06
Ammonia recycling	18	1.31	9	9.23E-07	7.20E-05	2.46E-05
Alanine metabolism	6	0.437	4	0.000343	0.0264	0.00686
Aspartate metabolism	12	0.874	5	0.000925	0.0703	0.0123
Glucose-alanine cycle	12	0.874	5	0.000925	0.0703	0.0123
Galactose metabolism	25	1.82	7	0.0013	0.0961	0.0143
Malate-aspartate shuttle	8	0.583	4	0.00143	0.104	0.0143
Arginineand prolinemetabolism	26	1.89	7	0.00168	0.121	0.0149
Citric acid cycle	23	1.67	6	0.00441	0.313	0.0353

**Fig. 1.**
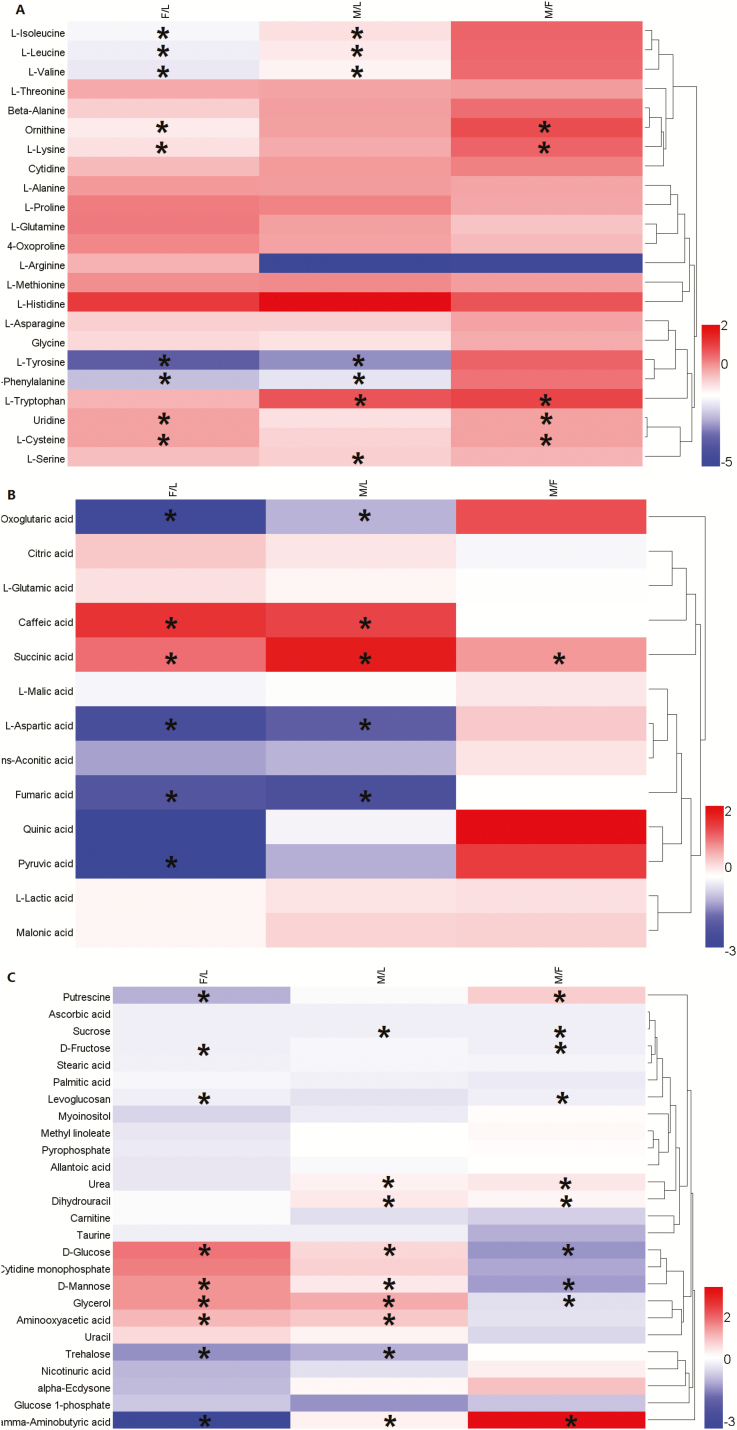
Comparison of the metabolome in paurometabolous development of *C. ciliata*. Heatmaps represent log2 fold change (adult females/ nymphs [F/L], adult males/ nymphs [M/L] and adult males/ adult females [M/F]) of the (A) amino acids, (B) organic acids, (C) other metabolites of *C. ciliata*. Asterisk indicate significant differences (P < 0.05).

**Fig. 2.**
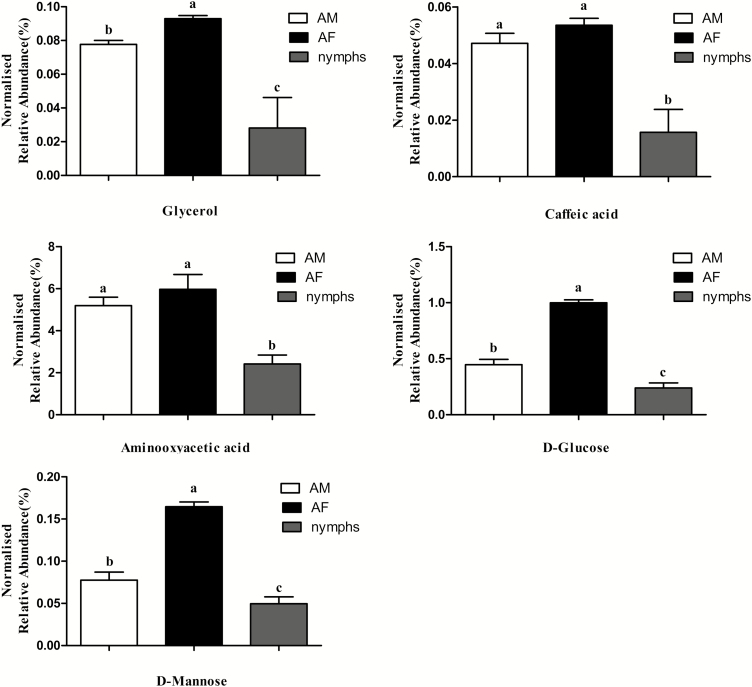
Metabolite abundances among nymphs, AM and AF.

To analyze changes in metabolites during development of *C. ciliata*, we measured and compared the metabolic profiles of nymphs, AM, and AF. Among the 62 metabolites identified in this study, 29 metabolites showed differences among nymphs, AM and AF (Fig. 1). Among them, five metabolites including one organic acid (caffeic acid), two sugars (D-glucose and D-mannose), one polyol (glycerol), and one other metabolite (aminooxyacetic acid), were significantly increased in content from nymphs to both adults (male and female), and nine metabolites significantly decreased in the paurometabolous development. D-glucose, D-mannose, and glycerol in AM were significantly higher than those in AF (Fig. 2).

### AKR Identification, Gene Cloning, and Phylogenetic Analysis

**Fig. 3.**
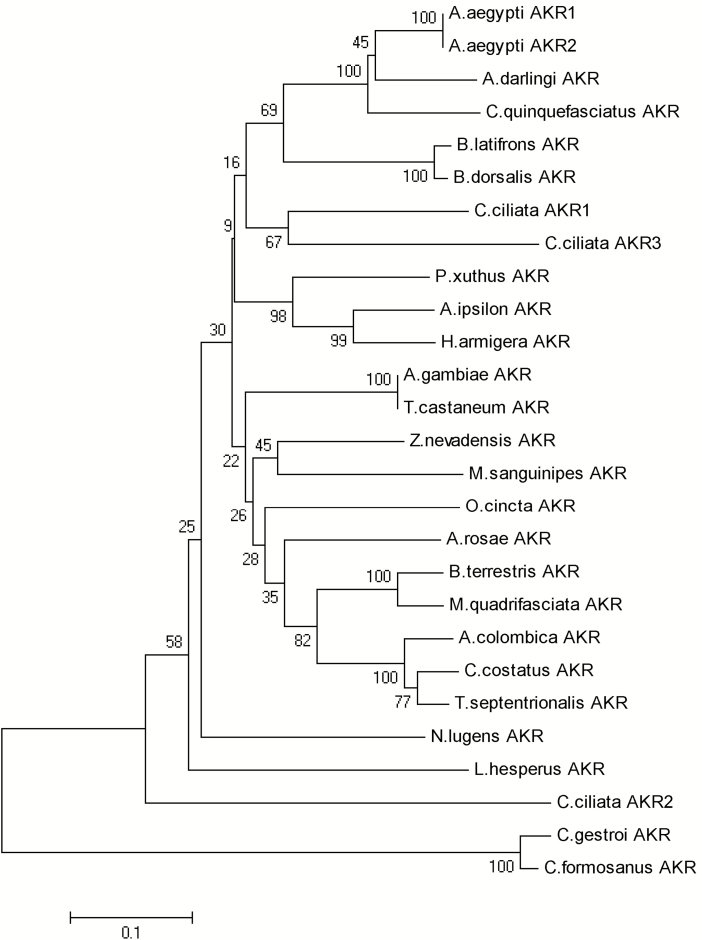
Phylogenetic analysis of aldo-keto reductase genes.

**Fig. 4.**
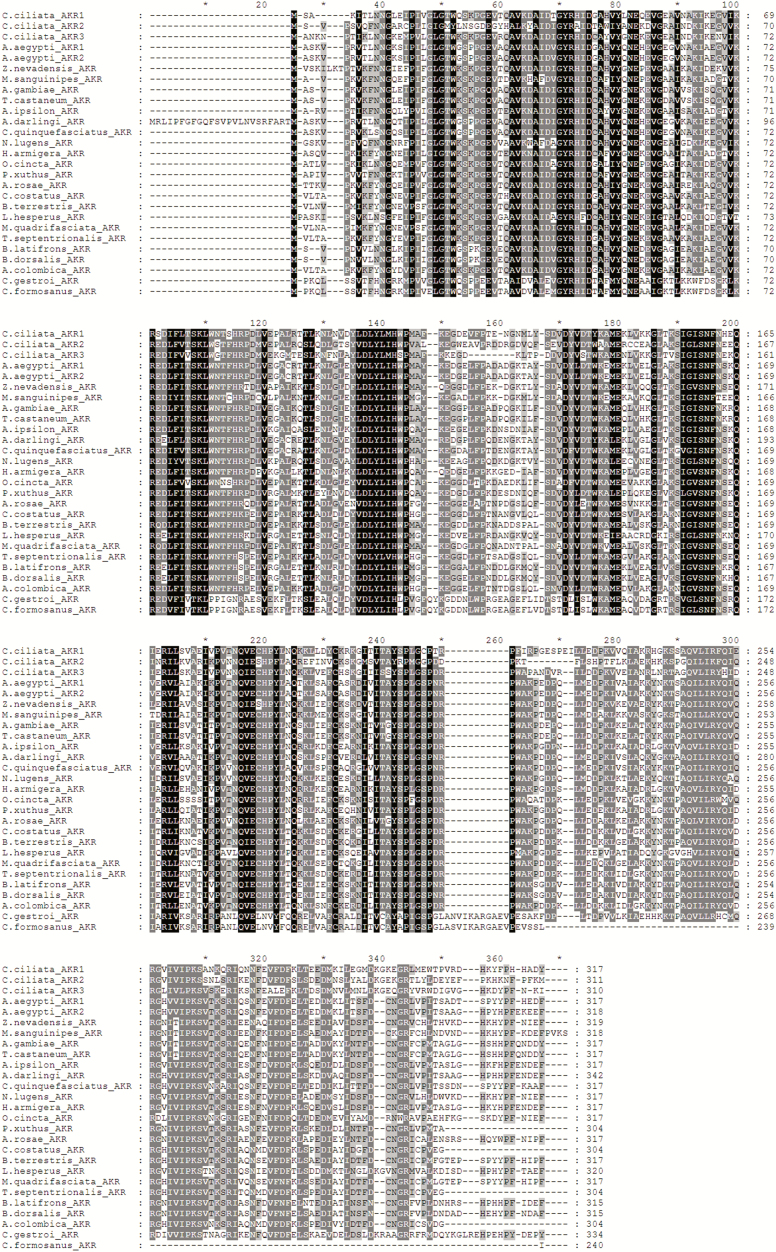
Multiple sequence alignment of the AKR superfamily. Black shading indicates 100% identity, dark-gray shading indicates 80–100% identity and light-gray shading indicates 60–80% identity.

Three AKR genes (c33223_g1, c57167_g1, and c30054_g1) were identified from RNA-seq datasets (Li et al. 2016). PCR was performed to clone the complete ORFs of the genes. The protein sequences of *C. ciliata* AKR1 (c33223_g1), AKR2 (c57167_g1), and AKR3 (c30054_g1) were phylogenetically analyzed by MEGA5. The phylogenetic tree showed that *C. ciliata* AKR1 had the closest relationship with AKR3, while *C. ciliata* AKR2 had the closest relationship with AKR (XP_022196473.1) from the brown planthopper *Nilaparvata lugens* (Stål) (Hemiptera: Delphacidae) (Fig. 3). The identity between *C. ciliata* AKR1 and AKR3 was 63%. The identity between *C. ciliata* AKR2 and *N. lugens* AKR was 55%. The amino acid sequence alignment of *C. ciliata* AKR with other known AKRs shown conserved motifs and domains typical of AKR (Fig. 4). All three *C. ciliata* AKRs contained the catalytic tetrad (Asp^75^, Tyr^80^, Lys^109^, and His^142^) characteristic of other AKRs (Jez et al. 1997). Moreover, all three *C. ciliata* AKRs annotated to glycerolipid metabolism, and involved in the metabolism of glycerol.

**Fig. 5.**
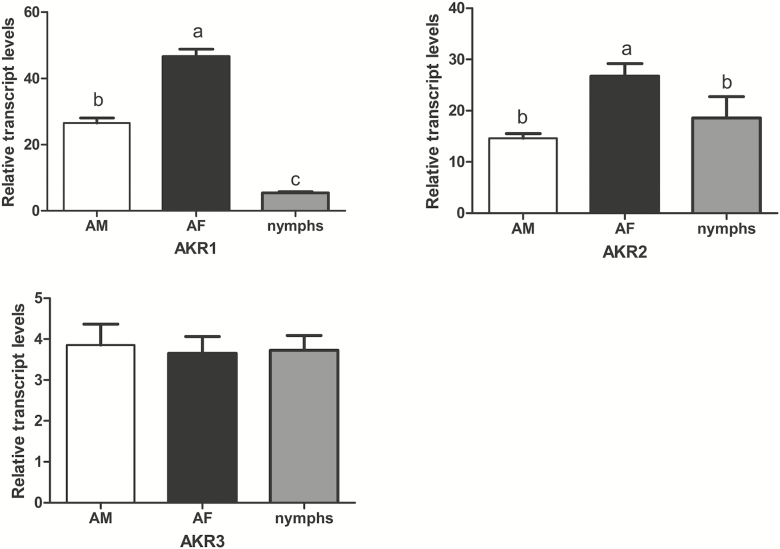
qRT-PCR validation of AKR in *C. ciliate.*

In order to verify the reliability of transcriptional expression levels in the transcriptome data, AKR1, AKR2, and AKR3 expression were further examined by qRT-PCR. The expression of AKR1 in AF was significantly up-regulated than that in AM, and in AM was significantly greater than that in nymphs. The expression of AKR2 in AF was significantly higher than that in both AM and nymphs. There were no significant differences in the expression of AKR3 among the three groups (Fig. 5).

## Discussion

The majority of metabolome studies have utilized the model organism *Drosophila melanogaster* Meigen (Diptera: Drosophilidae) (Hammad et al. 2011, Al Bratty et al. 2012, Colinet and Renault 2012). Our results highlight for the first time the metabolomics profiling and AKR characterization of *C. ciliate* during paurometabolous development. Very few data were available to date on the effect of metabolites changes on paurometabolous development of insect species from the *Corythucha* genus. The physiological needs of insects at different stages of development are different. Their gene expression levels and metabolites will also change accordingly. *Corythucha ciliata* is a paurometabolous insect. Both adults and nymphs feed on the foliage of sycamore trees. But only adult can overwinter under the loose bark of the host tree. In this study, we analyzed the metabolite profiles of this pest and combined the analysis of transcriptomes previously (Li et al. 2016), attempted to understand the changes in metabolites in *C. ciliata* during paurometabolous development.

Many animals possess a distinct immature developmental form (e.g., in case of insects nymph forms are distinct during the developmental period). The immature forms are much more adapted to environmental conditions than adults and consume more food to undergo the process of transition from immature to adult form. Nymph stages undergo metamorphosis in which they usually change in shape, size, and organization to form an adult. These changes are triggered by the alteration in metabolites during the development (Kaleka et al. 2019).

The AKRs belong to the NADP-dependent oxidoreductase superfamily, which play important roles in various physiological functions in prokaryotic and eukaryotic organisms. However, many AKR superfamily members remain uncharacterized in insect. Recent research has shown that the AKR AKR2E4 reduces 3-dehydroecdysone to ecdysone in the silkworm *Bombyx mori* L. (Lepidoptera: Bombycidae) (Yamamoto et al. 2017). In this study, we found that glycerol content in both adult groups were significantly higher than that in nymphs, and the AKR genes (c33223_g1 and c57167_g1) associated with glycerol were also significantly up-regulated in adults compared to that in nymphs. Glycerol is a cryoprotectant that can facilitate insect survival in low temperatures (Lee 1991). Adults of *C. ciliata* can bear acutely low temperatures in the winter and spring seasons in China. Therefore, our observation of greater content of glycerol and up-regulation of AKRs in adults than in nymphs may explain why *C. ciliata* adults can and nymphs cannot overwinter. Furthermore, glycerol is an important substrate of gluconeogenesis. It can be converted into glycerol 3-phosphate by phosphorylation and metabolized to D-glyceraldehyde by AKR undergoing re-esterification to form triacylglycerols (Hyndman et al. 2003, Hagopian et al. 2008). In the adult stage, insects consume more energy during the metabolic processes required for movement, flight, and reproduction (El-Shesheny et al. 2016).

The functions of the other four significantly increased metabolites are not as well understood as is the functions of glycerol in insects. The published studies available report caffeic acid as an active antioxidant, which might have beneficial effects on health in vivo (Gülçin 2006). Both D-glucose and D-mannose are monosaccharides. It is well known that D-glucose can be converted from glycerol through gluconeogenesis (Robergs and Griffin 1998).

The AKR family is a superfamily which contains more than 190 members expressed in prokaryotes and eukaryotes (Hyndman et al. 2003, Penning 2015). Most of the research on AKRs is about mammals, and are considered drug targets (Penning et al. 2018). The information of AKRs on insects is limited. AKRs participate in a variety of metabolic pathways, including the metabolism of glycerol(Jeffery and Jörnvall 1983), According to the RNA-seq datasets, we identified three AKR genes (c33223_g1, c57167_g1, and c30054_g1). All are associated with glycerol in glycerolipid metabolism, and two of them (c33223_g1 and c57167_g1) exhibited increasing trends in expression levels similar to the increasing glycerol content from nymphs to adults. Further research on the function of AKR genes need to be performed using robust molecular techniques such as RNA interference (Huvenne and Smagghe 2010).

### Conclusions

Our metabolite datasets revealed the changes in metabolites content during paurometabolous development from nymphs to adults in *C. ciliata*. Combining data from previous studies (Li et al. 2016) with our metabolome analysis, will help us to understand the physiological mechanisms of *C. ciliata* during paurometabolous. These information can be used to promote the development of environmentally friendly approaches to disrupt the processes of metamorphosis, Moreover, the present study informs our understanding of the essential genes involved in glycerol formation in insects, however, the genes of other important differential compounds have yet to be investigated and, therefore, should be explored in future studies.

## Supplementary Material

iez117_suppl_Supplementary-Materials
