# Supplementary material for: Metabolomics Profiling and AKR Characterization During Paurometabolous Development of Corythucha ciliata (Hemiptera: Tingidae)
Source: J Insect Sci. 2019 Dec 9;19(6):16. doi: 10.1093/jisesa/iez117 (PMC6901127; doi:10.1093/jisesa/iez117)
Supplement: iez117_suppl_Supplementary-Materials [file iez117_suppl_supplementary-materials.docx]

Table S1. NCBI accession numbers for amino acid sequences in phylogenetic tree

| **Species** | **Gene name** | **Accession number** |
| --- | --- | --- |
| *Aedes aegypti* | 1. aegypti AKR1 | XP_001648462.1 |
|  | 1. aegypti AKR2 | XP_001648461.1 |
| *Zootermopsis nevadensis* | Z. nevadensis AKR | KDR07716.1 |
| *Melanoplus sanguinipes* | M. sanguinipes AKR | ALX00029.1 |
| *Anopheles gambiae* | 1. gambiae AKR | XP_308086.3 |
| *Tribolium castaneum* | T. castaneum AR | EEZ98895.1 |
| *Agrotis ipsilon* | 1. ipsilon AKR | AGQ45614.1 |
| *Anopheles darlingi* | A.darlingi AKR | ETN61456.1 |
| *Culex quinquefasciatus* | C. quinquefasciatus AR | XP_001844836.1 |
| *Nilaparvata lugens* | N. lugens SF | APA34037.1 |
| *Helicoverpa armigera* | H. armigera AKR | AEB26313.1 |
| *Orchesella cincta* | O. cincta AKR | ODM90218.1 |
| *Papilio xuthus* | P. xuthus AKR | KPI99254.1 |
| *Athalia rosae* | 1. rosae AR | XP_012256159.1 |
| *Cyphomyrmex costatus* | C. costatus AD | KYN03393.1 |
| *Bombus terrestris* | 1. terrestris AR | XP_012172367.1 |
| *Lygus hesperus* | L. hesperus AR | JAG01530.1 |
| *Melipona quadrifasciata* | M. quadrifasciata AR | KOX80214.1 |
| *Trachymyrmex septentrionalis* | T. septentrionalis AD | KYN45372.1 |
| *Bactrocera latifrons* | B. latifrons AR | JAI20250.1 |
| *Bactrocera dorsalis* | B. dorsalis AKR | JAC41401.1 |
| *Atta colombica* | A. colombica AD | KYM87328.1 |
| *Coptotermes gestroi* | C. gestroi AKR | AMJ21949.2 |
| *Coptotermes formosanus* | C. formosanus AKR | AGM32584.1 |

Table S2. Primers used in this study for qRT-PCR validation

| unigene name | Forward primer | Reverse primer |
| --- | --- | --- |
| c33223_g1 | TCAAGGAGGGCGTCATTAAAC | GCCAGTGCATCAAGTACAAATC |
| c57167_g1 | CGAATCAAACCGGTCAACAATC | GGCTGTTACCGACATACCTTT |
| c30054_g1 | GAGTGCCACCCTTACCTAAAC | CCAAAGGGCTGTAGGAACTAAT |
| actin | GGGTATGGAATCTTGCGGTATC | TGTTGGCGTACAGGTCTTTC |

Table S3. The metabolites identified by GC-MS

| Metabolite Name | KEGG ID | Metabolite Name | KEGG ID |
| --- | --- | --- | --- |
| L-Isoleucine | C00407 | L-Leucine | C00123 |
| L-Valine | C00183 | L-Threonine | C00188 |
| L-Alanine | C00041 | Beta-Alanine | C00099 |
| L-Lysine | C00047 | Putrescine | C00134 |
| L-Proline  L-Arginine | C00148  C00062 | L-Glutamine  L-Methionine | C00064  C00073 |
| 4-Oxoproline | C01877 | Oxoglutaric acid | C00026 |
| Citric acid  Succinic acid  Ascorbic acid  Glycine  L-Tyrosine  L-Tryptophan  D-Glucose  Glucose 1-phosphate  Cytidine  L-Serine  L-Glutamic acid  D-Mannose  Glycerol  Levoglucosan  Palmitic acid  L-Aspartic acid  Stearic acid  Carnitine  Quinic acid  Urea  Caffeic acid  Ecdysone  Aminooxyacetic acid  Pyruvic acid | C00158  C00042  C00072  C00037  C00082  C00078  C00031  C00103  C00475  C00065  C00025  C00159  C00116  -  C00249  C00049  C01530  C00487  C06746  C00086  C01481  C00477  -  C00022 | L-Malic acid  Fumaric acid  L-Asparagine  L-Histidine  L-Phenylalanine  L-Lactic acid  Trehalose  Uridine  Cytidinemonophosphate  Ornithine  D-Fructose  Sucrose  Myoinositol  L-Cysteine  Gamma-Aminobutyricacid  Methyl linoleate  Pyrophosphate  Taurine  Uracil  Nicotinuric acid  Malonic acid  Dihydrouracil  Allantoic acid  trans-Aconitic acid | C00149  C00122  C00152  C00135  C00079  C00186  C01083  C00299  C00055  C00077  C02336  C00089  C00137  C00097  C00334  -  C00013  C00245  C00106  C05380  C00383  C00429  C00499  C02341 |

- means no hit in KEGG
